# Preliminary Evidence That the Short Allele of 5-HTTLPR Moderates the Association of Psychiatric Symptom Severity on Suicide Attempt: The Example in Obsessive-Compulsive Disorder

**DOI:** 10.3389/fpsyt.2022.770414

**Published:** 2022-04-01

**Authors:** Ghina Harika-Germaneau, Claire Lafay-Chebassier, Nicolas Langbour, Bérangère Thirioux, Issa Wassouf, Xavier Noël, Nemat Jaafari, Armand Chatard

**Affiliations:** ^1^Unité de Recherche Clinique Pierre Deniker, Centre Hospitalier Henri Laborit, Poitiers, France; ^2^Laboratoire de Neurosciences Expérimentales et Cliniques, Université de Poitiers, Poitiers, France; ^3^Centre de Recherches Cognition et Apprentissage, CNRS 7295, Université de Poitiers, Poitiers, France; ^4^Service de Pharmacologie Clinique et Vigilances, CHU de Poitiers, Poitiers, France; ^5^Laboratoire de Psychologie Médicale et d’Addictologie, ULB Neuroscience Institute, CHU Brugmann, Université Libre de Bruxelles, Brussels, Belgium

**Keywords:** severity of symptoms, serotonin transporter gene polymorphism, endogenous stress factor, gene-by-symptoms interaction, suicide attempts

## Abstract

**Background:**

The severity of symptoms represents an important source of distress in patients with a psychiatric disease. However, the extent to which this endogenous stress factor interacts with genetic vulnerability factors for predicting suicide risks remains unclear.

**Methods:**

We evaluated whether the severity of symptoms interacts with a genetic vulnerability factor (the serotonin transporter gene-linked promoter region variation) in predicting the frequency of lifetime suicide attempts in patients with a psychiatric disease. Symptom severity and 5-HTTLPR polymorphism were collected from a sample of 95 patients with obsessive-compulsive disorder (OCD). Lifetime suicide attempt was the primary outcome, and antecedent of multiple suicide attempts was the secondary outcome.

**Results:**

The gene-by-symptoms interaction was associated with an excess risk of suicide attempts (OR = 4.39, 95CI[1.44, 13.38], *p* < 0.009) and of multiple suicide attempts (OR = 4.18, 95CI[1.04, 16.77], *p* = 0.043). Symptom severity (moderate, severe, or extreme) was associated with an approximately five-fold increase in the odds of a lifetime suicide attempt in patients carrying one or two copies of the short allele of 5-HTTLPR. No such relationship was found for patients carrying the long allele.

**Conclusion:**

This study provides preliminary evidence for the gene-by-stress interaction on suicide attempt when stress is operationalized as symptom severity. Progress in suicide research may come from efforts to investigate the gene-by-symptoms interaction hypothesis in a variety of diseases.

## Introduction

Suicide rates have increased substantially over the last two decades (1–3), and the COVID-19 pandemic may worsen this health crisis (4, 5), although it has not yet been fully clarified in which modalities (both with regard to the temporal characteristics in relation to the different phases of the pandemic and with regard to the types of populations most at risk). Thus, it is important to understand the determinants of suicidal behavior (SB). Even if the causes of SB are not yet well understood (6), suicides are often associated with an underlying psychiatric disease. Indeed, a mental disorder is associated with an approximately eight-fold increase in the risk of suicide (7). However, only a minority of individuals with a psychiatric disease is likely to attempt suicide (7). This suggests that a psychiatric disease, and the psychological distress associated with it, is not sufficient by itself for explaining SB. A few genetic vulnerability factors can contribute to explaining why certain patients attempt to take their lives, while others will not. Understanding how the psychological distress associated with a psychiatric disease interacts with genetic factors to increase the vulnerability to suicide is both an important scientific aim and a pressing health issue.

One genetic vulnerability factor that has received substantial attention in the past is the serotonin transporter gene (SLC6A4 or 5-HTT). Abnormalities in the serotonergic system have long been considered a biomarker of SB (8). The serotonin transporter-linked polymorphic region (5-HTTLPR), consisting of a 44-pb deletion/insertion in the promotor region, regulates the serotonergic neurotransmission by influencing the reuptake of serotonin (5-HT). In-vitro studies (9) have indicated that the short (S) allele of the 5-HTTLPR is associated with lower transcriptional efficacy compared with the long (L) allele. Thus, the S-allele is related to a reduced availability of serotonin transporter in the synaptic cleft. In-vivo studies further showed an association of 5-HTTLPR with the efficacy of selective serotonin reuptake inhibitors (SSRIs) in depressed patients (10–12). However, 5-HTTLPR may not play a direct causal role in depression and suicide (13–15). Rather, serotonin (5-HT) neurotransmission appears to increase stress sensitivity and make individuals more vulnerable to negative effects (16). For example, in a large-scale prospective longitudinal study, Caspi et al. (17) found that 5-HTTLPR interacts with stressful life events to predict the risk of depression and suicide attempts (SA). Stressful life events had a stronger impact on the risk of depression and suicide in individuals with one of two copies of the S-allele of 5-HTT, compared to those with only the L-allele. Moreover, a number of functional magnetic resonance imaging (fMRI) studies have found that carriers of the S-allele show increased amygdala activation in response to unpleasant stimuli, thereby indicating increased stress sensitivity (18). Thus, the S-allele of 5-HTT may confer a genetic vulnerability for depression and suicide by exacerbating the negative effects of stress through increased amygdala activation.

However, the extant scientific literature indicates mixed findings, and the replicability of the 5-HTTLPR by stress interaction hypothesis is a controversial issue. In particular, two initial meta-analyses (19, 20) found no evidence that 5-HTTLPR moderates the relationship between stress and depression. Another meta-analysis by Karg et al. (21), which includes a larger number of studies (*n* = 56), provided more supportive results. However, two longitudinal studies that utilized rather large samples (over 4,000 individuals) found no interaction between 5-HTTLPR and stressful events for risk of depression and suicidality (22, 23). Moreover, a recent genome-wide association (GWA) study using data from large population-based samples (with Ns ranging from 62,138 to 443,264 across subsamples) found no evidence to support moderation of polymorphism effects by exposure to traumatic events on depression (24). In the same way, a recent collaborative meta-analysis (total *N* = 38,802) finds no evidence of a strong interaction between stress (negative life events) and 5-HTTLPR genotype contributing to the development of depression (25). Overall, the accumulated evidence casts doubt on the hypothesis that there is a robust gene × environment interaction involving 5-HTTLPR and stress to predict the risk of depression and suicide.

Given the actual state of the art, it is tempting to suggest that the gene by stress interaction is wrong and that researchers should stop wasting time and effort to test this hypothesis [(24); but see (26)]. However, it may be premature to draw such a conclusion since it is yet possible that an interaction exists in some limited situations (25). It is noteworthy, for instance, that prior studies have mainly considered exogenous stressors such as negative life events, traumatic events or childhood adversity as moderators. This operational definition of stress provides a stringent test of the gene by environment interaction hypothesis. However, stress may also stem from more endogenous factors such as symptom severity in psychiatric disease. In effect, psychiatric diseases are characterized by a specific set of symptoms that constitutes a major source of stress for patients, particularly when the symptoms are severe and persistent. Since most psychiatric diseases arise from combinations of genetic and environmental factors, it is obvious that symptom severity constitutes an endogenous stressor different from the purely environmental stressors that have been considered in prior research.

Somewhat surprising, little effort has been made thus far to examine whether the severity of psychiatric symptoms moderates the association between the serotonin transporter gene (5-HTTLPR) polymorphism and suicide in a psychiatric disease. The present study aimed to redress this neglect. Using a sample of patients treated for several years in our clinical unit for obsessive-compulsive disorder (OCD), we tested whether the 5-HTTLPR polymorphism moderates the relationship between symptom severity and suicide. We expected OCD symptom severity to be associated with an excess risk of suicide, mainly or only among carriers of the short allele of the 5-HTTLPR polymorphism.

## Materials and Methods

### Study Population

Ninety-five OCD outpatients were included in this study. Only native ethnic French outpatients, over 18 years of age, with a current OCD (according to The Diagnostic and Statistical Manual of Mental Disorders DSM-IV criteria, with Yale-Brown Obsessive-Compulsive Scale (Y-BOCS) > 16) were included. The exclusion criteria were actual depression, psychotic disorders, current substance abuse or dependence (except tobacco), developmental delay, severe organic or neurological pathology, pregnancy, and breastfeeding.

All patients were recruited over an eight-year period (from 2010 to 2018) from the Department of Psychiatry, Poitiers Henri Laborit Psychiatric Hospital (France). Poitiers is a midsize city in south-west France. We included all eligible patients treated in our clinical unit who volunteered to take part in this study during this period. Thus, it was not possible to include a larger sample of patients during this 8-year period. The expected effect size was unknown and the sample size was not informed by an *a priori* power analysis. However, a sensitivity analysis showed that this sample size provided adequate power (1 – β = 0.80) to detect a significant effect (*p* < .05) with an odds ratio of 2.14 or greater.

The institutional ethical committee approved the study protocol. All participants gave their informed written consent for study participation and for genetic analyses. All the procedures were performed in accordance with relevant guidelines and regulations.

### Clinical Assessment

Clinical assessments were performed blind to genotyping results. Clinical assessments included structured psychiatric interviews and sociodemographic characteristics. The number of SA was determined through direct questions regarding the implementation of the act during an in-depth interview with each patient. Patients reported the presence of SA at some point in his or her life. Lifetime SA was coded 1 for yes and 0 for no. The number of SA was heavily skewed and was, thus, recoded into a dichotomous variable: multiple SA coded 1 for more than 1 SA and 0 for less than 1 SA. Multiple SA has long been considered as a risk factor for suicide. There were 25 patients (23.7%) with a lifetime SA and 13 patients (13.6%) with a multiple SA.

The lifetime major depressive disorder was assessed by module A of the Mini International Neuropsychiatric Interview (MINI). The lifetime presence of trauma or post-traumatic stress disorder (PTSD) was assessed by module I of MINI. The severity of actual depression was assessed with the MADRS. Finally, the severity of OCD was assessed by the Y-BOCS, that is, one of the most commonly used structured-interview methods to assess OCD symptoms. The Y-BOCS is a 10-item scale designed to measure the severity and type of symptoms (obsessions and compulsions) in people with OCD over the past 7 days. The scale is used to assess obsessions (5 items; e.g., “How much of your time is occupied by obessive thoughts?”; 0 = None, 4 = Greater than 8 h/day or nearly constant occurrence) and compulsions (5 items; e.g., “How much do your compulsive behaviors interfere with your work, school, social, or other important role functioning? Is there anything that you don’t do because of the compulsions?”; 0 = None, 4 = Incapacitating). The Y-BOCS is a well-validated instrument, which has satisfactory convergent validity, high internal consistency and high interrater reliability (27). Total Y-BOCS scores range from 0 to 40, with higher scores indicating greater severity of OCD symptoms. Scores on the obsession and compulsion subscales range from 0 to 20, but only the total Y-BOCS score is interpreted. Total scores can be split into five categories, based on severity of symptoms: subclinical (0 to 7), mild (8–15), moderate (16–23), severe (24–31), and extreme case of OCD (32–40). The present sample included only patients with moderate (*n* = 6, 6.3%), severe (*n* = 65, 68.4%), or extreme (*n* = 24, 25.2%) scores. The mean of OCD symptoms (total scores) was 28.5 (SD = 3.97).

### Genotyping

Genomic DNA was extracted from the peripheral EDTA-anticoagulated blood mononuclear cells using an automated nucleic acid extraction system (Magtration System 12GC Bionobis) and was stored at −20°C. Restriction Fragment Length Polymorphism (RFLP) was utilized to determine the insertion/deletion of 44 base pairs (bp) of 5-HTTLPR (28). To implement this technique, we use the restriction enzyme MspI (Boehinger Mannheim). Since the direction primer is marked with a fluorochrome, one of the fragments obtained is visualized by capillary electrophoresis on the sequencer (Applied Biosystems 3130) and the size is determined by comparison with a size marker, which is also fluorescent. In order to perform the PCR, the following reaction mixture is used according to the manufacturer’s recommendations: KAPA2G GC 1X buffer, 0.25 mM dNTPs, 0.2 μM primers each, 0.2 units KAPA2G Taq polymerase, and 25 ng of DNA for a reaction volume of 12.5 μL. The tubes are then placed in the thermal cycler to undergo 30 PCR cycles. The program used includes a three-minute cycle of initial denaturation at 95°C, 30 cycles comprising 30 s of denaturation at 95°C, 30 s of hybridization at 60°C, and 30 s of extension at 72°C, and finally a last extension cycle of 5 min at 72°C. The direction primer is marked by a fluorochrome FAM (6-Carboxy Fluorescein, fluorescent in blue) fixed at the 5’end; the absorption peak of the direction primer is at 530 nm. One of the fragments obtained is visualized by capillary electrophoresis on the sequencer (Applied Biosystems 3130) and the size is determined by comparison with a size marker, which is also fluorescent.

The 5-HTTLPR genotypes were evaluated as blind samples. Each analysis included positive and negative controls for quality assurance. The genotyping for five patients could not be determined based on the extracted samples, leaving 90 patients in the analyses. The patients were classified into three genotypes: L/L, L/S, and S/S. However, consistent with Caspi et al. (17) original findings, patients carrying the long allele (L/L) were differentiated from those carrying one of two copies of the short allele (L/S and S/S) in the main analyses. There were 28 patients (30.3%) with the long (L/L) allele and 62 patients (69.6%) with one or two copies of the short allele (L/S or S/S) of the 5-HTT gene.

### Covariates

The control variables were gender, age, age-of-onset, lifetime major depressive disorder, and lifetime PTSD. These variables were considered given their associations with suicidal behavior in previous work.

### Statistical Analyses

Logistic regression analyses were employed to test the interaction between chronic symptoms and genes on SB. Lifetime SA was the primary outcome, and multiple SA was the secondary outcome. For the sake of transparency, we report results with and without control for covariates. The Hardy-Weinberg equilibrium (HWE) was checked in the entire sample and distribution of alleles was compared using a Chi-square test. The statistical significance level was fixed at 0.05.

## Results

### Preliminary Analyses

Table 1 shows the characteristics of the sample. The genotypic distribution of the 5-HTTLPR polymorphisms was found to be in HWE (*p* = 0.72). Bivariate analyses showed that OCD symptom severity (continuous variable) was positively correlated with lifetime SA (*r*_*pb*_ = .203, *p* = .048) and with multiple SA (*r*_*pb*_ = .200, *p* = .057). An analysis of variance (ANOVA) indicated that patients carrying the S and L allele of the 5-HTT gene did not differ in OCD symptom severity, *F*(1,87) = 1.06, *p* = .30, Cohen’s *d* = –.28, 95CI[−2.72, 0.86]. Overall, compared to carriers of the L allele, carriers of the S allele did not show a greater frequency of SA (OR = 1.43, 95CI[0.49, 4.13], *p* = 0.50) or multiple SA (OR = 1.35, 95CI[0.33, 5.44], *p* = 0.67).

**TABLE 1:** Characteristics of the sample.

Variable	All patients	L/L allele	5-HTTLPR L/S allele	S/S allele
Sex (%)				
Women	55.32	36.17	40.42	23.40
Men	44.68	26.19	52.38	21.42
Age (*M*, *SD*)	44.39 (15.24)	41.90 (18.74)	45.16 (13.45)	44.27 (15.24)
Age of onset of OCD (*M*, *SD*)	18.66 (10.99)	16.36 (7.12)	19.33 (11.42)	17.10 (9.26)
Clinical variables	49.50	30.23	53.48	16.27
Lifetime depressive disorder (%)	1.11	0.00	100.00	0.00
Lifetime PTSD (%)				
OCD symptoms (*M*, *SD*)				
Total score	28.54 (3.97)	27.86 (3.98)	29.14 (3.65)	27.80 (4.20)
Obsession score	14.37 (2.97)	13.64 (3.34)	14.60 (2.82	14.75 (2.69)
Compulsion score	14.61 (2.42)	14.21 (2.28)	15.07 (2.52)	14.05 (2.43)
OCD symptoms (%)				
Moderate (16–23)	6.30	60.00	40.00	0.00
Severe (24–31)	68.40	30.15	49.20	20.63
Extreme (32–40)	25.30	27.27	50.00	22.72
Lifetime suicide attempts (%)				
One previous attempt	26.30	25.00	58.30	16.70
Multiple previous attempts	14.30	25.00	66.70	8.30

*OCD = Obsessive-compulsive disorder. PTSD = post traumatic stress disorder.*

### Main Analyses

We begin by reporting the results of our main analyses. The probability of a lifetime SA, our primary outcome, was predicted from chronic OCD symptoms (total OCD scores, continuous variable), 5-HTTLPR genotyping (dichotomous variable), and the product term between chronic OCD symptoms and 5-HTTLPR. To facilitate interpretation of the odds ratio, chronic OCD symptoms were standardized such that an increase in one unit corresponded to an increase in one SD (i.e., approximately four points on the OCD scale). The odds ratio and 95%CI are depicted in Table 2. As indicated in this table, there were no significant associations between 5-HTTLPR and lifetime SA on the one hand and between OCD symptoms and lifetime SA on the other. However, the interaction between 5-HTTLPR variation and OCD symptoms was significant (OR = 4.39, 95CI[1.44, 13.38], *p* = 0.009). Whenever OCD symptoms increase by 1SD, the S-allele, compared to the L-allele, is associated with an approximate four-fold increase in the odds of reporting a lifetime SA.

**TABLE 2:** Logistic regression analysis predicting lifetime suicide attempt from 5-HTTLPR, chronic OCD symptoms, and the interaction between 5-HTTLPR and chronic OCD symptoms.

						95% Confidence interval	
Predictor	Estimate	SE	Wald	p	Odds ratio	Lower	Upper
Intercept	−1.417	0.517	7.518	0.006	0.242		
5-HTTLPR (S-allele)	0.328	0.610	0.288	0.591	1.388	0.420	4.587
Y-BOCS	−0.508	0.463	1.203	0.273	0.602	0.243	1.491
5-HTTLPR × Y-BOCS	1.480	0.568	6.782	0.009	4.393	1.442	13.382

*5-HTTLPR (S-allele) = Short allele of 5-HTT gene; YBOCS = standardized obsessive-compulsive symptoms (total scores).*

To gain further insight into the nature of this interaction, the odds of reporting a lifetime SA were estimated from the severity of OCD symptoms (moderate, severe, or extreme) separately for patients carrying the L-allele (L/L) and the S-allele (L/S or S/S). For patients carrying the L-allele, the severity of OCD symptoms was associated with a non-significant decrease in the odds of reporting a lifetime SA (OR = 0.63, 95CI[0.12, 3.26], *p* = 0.588). In contrast, for patients carrying the S-allele, the severity of OCD symptoms was associated with an approximate five-fold increase in the odds of reporting a lifetime SA (OR = 5.25, 95CI[1.59, 17.38], *p* = 0.007). Thus, extreme (severe) OCD symptoms, compared to moderate OCD symptoms, were associated with an approximate 10-fold (5-fold) increase in the odds of reporting a lifetime SA in patients carrying the S-allele.

In the same manner, the probability of multiple SA—our secondary outcome—was predicted from chronic OCD symptoms (total OCD scores, continuous variable), 5-HTTLPR genotyping (dichotomous variable), and the product term between chronic OCD symptoms and 5-HTTLPR. In this analysis, there was no association between OCD symptoms and multiple SA and no association between 5-HTTLPR and multiple SA (see Table 3). However, the interaction between chronic OCD symptoms and 5-HTTLPR variation was significant in predicting multiple SA (OR = 4.10, 95CI[1.03, 16.24], *p* = 0.044). Thus, whenever OCD symptoms increase by 1SD, the S-allele—compared to the L-allele— is associated with an approximate four-fold increase in the odds of reporting a multiple SA.

**TABLE 3:** Logistic regression analysis predicting multiple suicide attempts from 5-HTTLPR, chronic OCD symptoms, and the interaction between 5-HTTLPR and chronic OCD symptoms.

						95% Confidence interval	
Predictor	Estimate	SE	Wald	p	Odds ratio	Lower	Upper
Intercept	−2.116	0.680	10.156	0.001	0.115		
5-HTTLPR (S-allele)	0.031	0.831	0.001	0.971	1.031	0.202	5.260
Y-BOCS	−0.389	0.570	0.466	0.495	0.678	0.222	2.071
5-HTTLPR × Y-BOCS	1.412	0.702	4.046	0.044	4.104	1.037	16.245

*5-HTTLPR (S-allele) = Short allele of 5-HTT gene; YBOCS = standardized obsessive-compulsive symptoms (total scores).*

The odds of reporting a multiple SA were estimated from the severity of OCD symptoms (moderate, severe, or extreme) separately for patients carrying the L-allele (L/L) and S-allele (L/S or S/S). For patients carrying the L-allele, the severity of OCD symptoms was associated with a non-significant decrease in the odds of reporting multiple SA (OR = 0.67, 95CI[0.08, 5.51], *p* = 0.713). In contrast, for patients carrying the S-allele, the severity of OCD symptoms was associated with an approximate eight-fold increase in the odds of reporting a multiple SA (OR = 7.94, 95CI[1.71, 36.80], *p* = 0.008). Thus, extreme (severe) OCD symptoms, compared to moderate OCD symptoms, were associated with an approximate 16-fold (8-fold) increase in the odds of reporting a lifetime SA in patients carrying the S-allele.

### Supplementary Analyses

**FIGURE 1:**
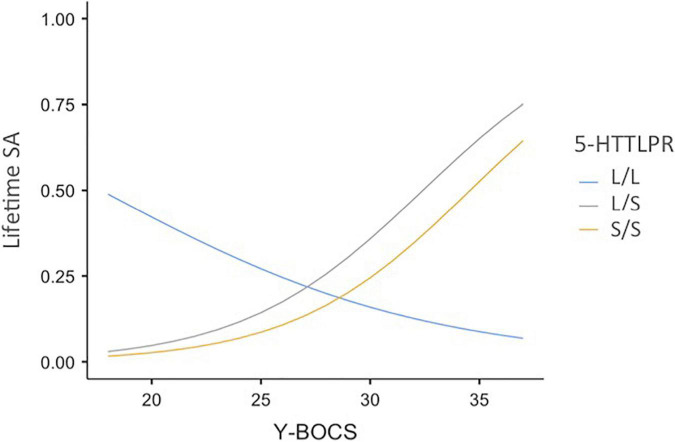
Probability of a lifetime suicide attempt as a function of OCD symptom severity and 5-HTTLPR genotyping variations. 5-HTTLPR = L/L, L/S, or S/S allele of 5-HTT gene; Y-BOCS = obsessive-compulsive symptoms (total scores); Lifetime SA = Probability of a lifetime suicide attempt.

In further analyses, we examined possible differences between the three genotypes (L/L, L/S, and S/S). The probability of a lifetime SA was predicted from chronic OCD symptoms (standardized total OCD scores), 5-HTTLPR genotyping (L/L, L/S, or S/S), and the product terms between chronic OCD symptoms and 5-HTTLPR. The odds ratio and 95% CI are presented in Table 4. As indicated in this table, the two interaction terms were significant. Whenever OCD symptoms increase by 1SD, the S/S (or L/S) allele—compared to the L/L allele—is associated with an approximate four-fold increase in the odds of reporting a lifetime SA. Figure 1 represents the probability of a lifetime suicide attempt estimated for the L/L, L/S, and S/S variations at different levels of OCD symptoms.

**TABLE 4:** Logistic regression analysis predicting lifetime suicide attempts from 5-HTTLPR, chronic OCD symptoms, and the interaction between 5-HTTLPR and chronic OCD symptoms.

						95% Confidence interval	
Predictor	Estimate	SE	Wald	p	Odds ratio	Lower	Upper
Intercept	−1.466	0.515	8.121	0.004	0.231		
5-HTTLPR:							
L/S – L/L	0.531	0.639	0.692	0.405	1.701	0.487	5.949
S/S – L/L	−0.021	0.827	0.001	0.979	0.979	0.194	4.950
Y-BOCS	−0.536	0.464	1.336	0.248	0.585	0.236	1.452
Y-BOCS × 5-HTTLPR:							
Y-BOCS × (L/S – L/L)	1.494	0.614	5.915	0.015	4.454	1.336	14.843
Y-BOCS × (S/S – L/L)	1.514	0.763	3.938	0.047	4.546	1.019	20.285

*5-HTTLPR = L/L, L/S, or S/S allele of 5-HTT gene; Y-BOCS = standardized obsessive-compulsive symptoms (total scores).*

In subsequent analyses, we examined whether the effects were similar after control for gender, age, age-of-onset, lifetime depression, lifetime PTSD, and severity of actual depression. We reproduced our main analyses after including these variables into the binomial logistic regression model to control for their effects. With regard to lifetime SA, the interaction between OCD symptom severity and 5-HTTLPR remained significant after control for these variables (OR = 6.59, 95CI[1.80, 24.13], *p* = 0.004). Among the control variables, only lifetime depression had a significant effect on lifetime SA (OR = 4.66, 95CI[1.33, 16.32], *p* = 0.016). On multiple SA, the interaction between OCD symptom severity and 5-HTTLPR was marginally significant after control for the covariates (OR = 5.59, 95CI[0.97, 32.22], *p* = 0.054). Among the control variables, only the effect of lifetime depression approached significance (OR = 6.38, 95CI[0.92, 43.94], *p* = 0.060).

Finally, we tested whether OCD symptom severity and 5-HTTLPR interacts to predict lifetime depression in a binomial logistic regression analysis. In this analysis, no significant association was found between 5-HTTLPR and lifetime depression (OR = 0.87, 95CI[0.33, 2.28], *p* = 0.783) on the one hand and no significant association was found between OCD symptoms and lifetime depression on the other hand (OR = 2.17, 95CI[0.85, 5.56], *p* = 0.105). Moreover, the interaction between chronic OCD symptoms and 5-HTTLPR was not significant for predicting lifetime depression (OR = 0.81, 95CI[0.27, 2.42], *p* = 0.707).

## Discussion

Why are patients with a psychiatric disease at greater risk for attempting suicide? We found evidence supporting the hypothesis that an endogenous factor of stress (chronic symptoms) interacts with a genetic vulnerability factor (variation of the serotonin transporter 5-HTTLPR polymorphism) to predict the lifetime prevalence of SB in patients with obsessive-compulsive disorders. Broadly, we found that OCD patients who were the most likely to report a lifetime SA, as well as multiple SA, were those who have both the most severe symptoms and one or two copies of the short allele of the serotonin transporter polymorphism. In this study, the symptoms × gene interaction was significant on lifetime SA, regardless of control variables (gender, age, age-at-onset, lifetime depression, lifetime post-traumatic stress disorder, and severity of actual depression). Similar findings were found on multiple SA, even though the effects were less pronounced and significant.

The present data are in line with previous research and theorizing, thereby suggesting that the primary motivation for suicide is intense psychological distress that eventually becomes intolerable and which cannot be escaped from by other means (e.g., treatment) than suicide (29, 30). Our data are also consistent with previous research that indicates that the 5-HTTLPR polymorphism in the serotonin transporter gene may contribute to the risk of SB under stress (15, 17, 31). Most research in this literature has focused on exogenous environmental factors of stress (adversity and negative life events). Our findings extend this line of research by indicating that an important endogenous factor of stress—the severity of chronic symptoms in patients with a psychiatric disease—interacts with the 5-HTTLPR polymorphism in the serotonin transporter gene for predicting the risk of suicide behavior. To the best of our knowledge, very few studies have sought to test this hypothesis. Yet, this relatively simple and straightforward hypothesis is potentially important as it may provide a parsimonious explanation for the high prevalence of suicide in individuals with a psychiatric disease.

The current study has a number of limitations. First, the sample was relatively small and homogeneous. The small sample size might account for some of the non-significant findings. For example, in the present sample, the overall association of the S allele of the 5-HTTLPR gene with SA was small and non-significant (OR = 1.38). This is consistent with previous research reporting no main effect of 5-HTTLPR on SA [e.g., (32)]. However, a main effect of the S allele of 5-HTTLPR on SA may be observable in limited situations, for example, in the case of violent suicide [see (32, 33)]. Further studies using larger samples of patients with OCDs are thus needed to test whether there is a direct association of the S allele of the 5-HTTLPR gene with violent suicide, independently from the severity of OCD symptoms.

In the present study, we focused on patients with OCDs. However, our reasoning is not restricted to this specific population. Further studies may examine the extent to which the present findings can be replicated in other clinical populations (patients with post-traumatic stress disorders, bipolar disorders, schizophrenia disorders, etc.) who are at risk for suicide. It would also be interesting to test whether the present findings can be extended to any other clinical populations suffering from non-psychiatric but severe and persistent symptoms (e.g., Parkinson’s disease).

Second, our main analyses contrasted between patients carrying the long allele (L/L) from those carrying one or two copies of the short allele (L/S and S/S). This was consistent with Caspi et al. (17) original work that revealed that carriers of one or two copies of the S-allele are at higher risk for suicide. However, a number of studies, including Caspi et al. (17) study, have also suggested that the S/S allele may contribute the most to SB. If the polymorphism functions through regulating gene expression (34), differences between L/S and S/S are to be expected. In the present study, however, we did not find support for this hypothesis. The relation between symptom severity and SA was similar in patients carrying the S/S and L/S allele. The small sample size may have contributed to this result. Further studies using much larger and representative samples are required to test allelic differences in the susceptibility to suicidal behavior.

Another limitation is the limited ability of the traditional candidate-gene approach to account for complex disorders such as suicidality. Such disorders are most likely the product of an interaction of multiple genes and environmental factors. A recent integrative model (35) suggests that the serotoninergic system does not operate in isolation but rather in interaction with the dopaminergic and noradrenergic systems to predict suicide risk in response to stressors. According to this model, dysregulation of the serotonin levels in the brain can influence behaviors such as impulsivity, decision-making, cognitive rigidity, and implicit association. Impaired dopaminergic functioning may further increase the risk of suicide by affecting the same cognitive and personality factors. The noradrenergic system may influence mood factors such as pessimism and hopelessness. Altogether, these different neurochemical systems can combine to increase the risk of suicide. Of course, the candidate gene approach is limited by how much is known of the neurobiology of SB. Even if recent models integrating various genetic and epigenetic markers for suicide show promising (35), it remains important to study the role of specific genes to better elucidate their respective role (26, 36). As Moore [(26), p. 331] noticed: “*Both candidate and whole genome strategies have limitations, and each approach is useful and valid in the quest to identify the elusive genetic architecture of complex behavioral phenotypes.*” (p. 331).

Finally, the current study relies on a cross-sectional design, which is limited in its ability to draw causal inferences. Future longitudinal studies with multiple waves may be useful to determine whether the gene × symptom interaction plays a causal role in suicidal behavior.

## Conclusion

The present results suggest that the severity of chronic symptoms is by itself a potent source of stress among patients with a psychiatric disease that may considerably increase odds of attempting suicide, especially when associated with the S allele of 5-HTTLPR. This finding has potentially important implications for the candidate-gene approach. As we have noticed, the results of prior gene-by-stress interaction studies have been inconsistent and contradictory. One possible explanation for that is that most studies have focused on some stressors that are only environmental, typically, a negative life event. In contrast, the present study relies on an endogenous stress factor –symptom severity–, which may be a better proxy of stress among patients with a psychiatric disease. The present findings support the possible contributory role of the S allele of 5-HTTLPR in the emergence of suicidality in patients struggling with severe psychiatric symptoms. Further studies testing the gene-by-symptoms interaction hypothesis with a variety of psychiatric diseases, large sample sizes, and a meta-analytic approach, may potentially lead to important progress in suicide research.

## Data Availability Statement

The original contributions presented in the study are included in the article/Supplementary Material, further inquiries can be directed to the corresponding author.

## Ethics Statement

The studies involving human participants were reviewed and approved by CPP. The patients/participants provided their written informed consent to participate in this study.

## Author Contributions

AC and GH-G conceptualized and designed the study, analyzed the data, and wrote the manuscript. CL-C provided assistance with genotyping. NL, BT, IW, and NJ provided help with data acquisition. NL checked data accuracy and analyses. All authors made critical intellectual revisions and substantive contributions to the manuscript.

## Conflict of Interest

The authors declare that the research was conducted in the absence of any commercial or financial relationships that could be construed as a potential conflict of interest.

## Publisher’s Note

All claims expressed in this article are solely those of the authors and do not necessarily represent those of their affiliated organizations, or those of the publisher, the editors and the reviewers. Any product that may be evaluated in this article, or claim that may be made by its manufacturer, is not guaranteed or endorsed by the publisher.

## Supplementary Material

Supplementary Table 1 Anonymized dataset.

Supplementary Data Sheet 1 Code for analyses.
