# Supplementary material for: Preliminary Evidence That the Short Allele of 5-HTTLPR Moderates the Association of Psychiatric Symptom Severity on Suicide Attempt: The Example in Obsessive-Compulsive Disorder
Source: Front Psychiatry. 2022 Apr 1;13:770414. doi: 10.3389/fpsyt.2022.770414 (PMC9010527; doi:10.3389/fpsyt.2022.770414)
Supplement: Supplementary Data Sheet 1 — Code for analyses. [file Data_Sheet_1.pdf]

```
*************Preliminary analyses

CORRELATIONS
  /VARIABLES=YBOCST TS_life MultipleSA
  /PRINT=TWOTAIL NOSIG
  /MISSING=PAIRWISE.

*************Main analyses Table 2 (primary outcome)

LOGISTIC REGRESSION VARIABLES TS_life
  /METHOD=ENTER AlleleS YBOCSTz AlleleS*YBOCSTz
  /CONTRAST (AlleleS)=Indicator(1)
  /PRINT=CI(95)
  /CRITERIA=PIN(0.05) POUT(0.10) ITERATE(20) CUT(0.5).


SORT CASES  BY AlleleS.
SPLIT FILE SEPARATE BY AlleleS.

LOGISTIC REGRESSION VARIABLES TS_life
  /METHOD=ENTER SymptomsCategory
  /PRINT=CI(95)
  /CRITERIA=PIN(0.05) POUT(0.10) ITERATE(20) CUT(0.5).

SPLIT FILE OFF.

*************Main analyses Table 3 (secondary outcome)

LOGISTIC REGRESSION VARIABLES MultipleSA
  /METHOD=ENTER AlleleS YBOCSTz AlleleS*YBOCSTz
  /CONTRAST (AlleleS)=Indicator(1)
  /PRINT=CI(95)
  /CRITERIA=PIN(0.05) POUT(0.10) ITERATE(20) CUT(0.5).


SORT CASES  BY AlleleS.
SPLIT FILE SEPARATE BY AlleleS.

LOGISTIC REGRESSION VARIABLES MultipleSA
  /METHOD=ENTER SymptomsCategory
  /PRINT=CI(95)
  /CRITERIA=PIN(0.05) POUT(0.10) ITERATE(20) CUT(0.5).

SPLIT FILE OFF.


*************Supplementary analyses Table 4

LOGISTIC REGRESSION VARIABLES TS_life
```

```
  /METHOD=ENTER Gene5HTTLPR YBOCSTz Gene5HTTLPR*YBOCSTz
  /CONTRAST (Gene5HTTLPR)=Indicator(1)
  /PRINT=CI(95)
  /CRITERIA=PIN(0.05) POUT(0.10) ITERATE(20) CUT(0.5).


*************Supplementary analyses (with covariates)

LOGISTIC REGRESSION VARIABLES TS_life
  /METHOD=ENTER Sex Age AgeofOnset LifetimeDepression PTSDPast MADRS AlleleS
YBOCSTz
    AlleleS*YBOCSTz
  /PRINT=CI(95)
  /CRITERIA=PIN(0.05) POUT(0.10) ITERATE(20) CUT(0.5).

LOGISTIC REGRESSION VARIABLES MultipleSA
  /METHOD=ENTER Sex Age AgeofOnset LifetimeDepression PTSDPast MADRS AlleleS
YBOCSTz
    AlleleS*YBOCSTz
  /PRINT=CI(95)
  /CRITERIA=PIN(0.05) POUT(0.10) ITERATE(20) CUT(0.5).
```
